# The effect of elimination of gibbs ringing, noise and systematic errors on the DTI metrics and tractography in a rat brain

**DOI:** 10.1038/s41598-024-66076-z

**Published:** 2024-07-01

**Authors:** Weronika Mazur-Rosmus, Artur T. Krzyżak

**Affiliations:** AGH University of Krakow, Al. Mickiewicza 30, 30-059 Krakow, Poland

**Keywords:** Diagnostic markers, Preclinical research

## Abstract

Diffusion tensor imaging (DTI) metrics and tractography can be biased due to low signal-to-noise ratio (SNR) and systematic errors resulting from image artifacts and imperfections in magnetic field gradients. The imperfections include non-uniformity and nonlinearity, effects caused by eddy currents, and the influence of background and imaging gradients. We investigated the impact of systematic errors on DTI metrics of an isotropic phantom and DTI metrics and tractography of a rat brain measured at high resolution. We tested denoising and Gibbs ringing removal methods combined with the B matrix spatial distribution (BSD) method for magnetic field gradient calibration. The results showed that the performance of the BSD method depends on whether Gibbs ringing is removed and the effectiveness of stochastic error removal. Region of interest (ROI)-based analysis of the DTI metrics showed that, depending on the size of the ROI and its location in space, correction methods can remove systematic bias to varying degrees. The preprocessing pipeline proposed and dedicated to this type of data together with the BSD method resulted in an even − 90% decrease in fractional anisotropy (FA) (globally and locally) in the isotropic phantom and − 45% in the rat brain. The largest global changes in the rat brain tractogram compared to the standard method without preprocessing (sDTI) were noticed after denoising. The direction of the first eigenvector obtained from DTI after denoising, Gibbs ringing removal and BSD differed by an average of 56 and 10 degrees in the ROI from sDTI and from sDTI after denoising and Gibbs ringing removal, respectively. The latter can be identified with the amount of improvement in tractography due to the elimination of systematic errors related to imperfect magnetic field gradients. Based on the results, the systematic bias for high resolution data mainly depended on SNR, but the influence of non-uniform gradients could also be seen. After denoising, the BSD method was able to further correct both the metrics and tractography of the diffusion tensor in the rat brain by taking into account the actual distribution of magnetic field gradients independent of the examined object and uniquely dependent on the scanner and sequence. This means that in vivo studies are also subject to this type of errors, which should be taken into account when processing such data.

## Introduction

Accurate determination of diffusion tensor imaging (DTI) metrics and tractography is desirable to reliably describe the microstructure in a quantitative manner. For example, in biological systems, altered microstructure may be an indicator of a lesion. One can find a huge number of studies in which DTI metrics, especially mean diffusivity (MD) and fractional anisotropy (FA), are proposed as biomarkers of a given abnormality (for example^1–4^). Therefore, accurate DTI metrics can positively impact diagnoses and medical research in general. However, DTI metrics are sensitive not only to the applied protocol, but also to stochastic and systematic errors, which are variable across the scanners and time. Most in vivo studies require preprocessing to correct for adverse effects which can be data-dependent. Therefore, many preprocessing pipelines have been proposed that are able to correct for the DTI metrics bias to a different extent^5–7^. These pipelines are also used in DTI, however, their limitation is the incomplete correction of the systematic bias associated with non-uniform magnetic field gradients by removing only the effects of eddy currents. In this work, we propose the additional step that could be incorporated in different DTI preprocessing pipelines to correct for the total systematic bias connected with non-uniform magnetic field gradient. The need for reliable (focus on accuracy) and reproducible (focus on precision) indices in biomedical imaging has been recognized and initiatives to meet this need were established^8^.

Systematic bias of DTI metrics can occur due to noise^9–19^, subject motions^20–22^ or imperfect magnetic field gradients^14,23–30^. Therefore, systematic errors can be reduced by increasing signal-to-noise ratio (SNR), but complete correction is unattainable even for high SNR^14,17^. The remaining part of the systematic error comes from the difference between the actual and desired (used in the calculations) diffusion gradient. The modification of the desired diffusion gradient is influenced by: imaging, background and residual gradients, eddy currents, spatial non-uniformity and nonlinearity of gradients, gradient scaling^23,25,26,29,30^. Another long unexplored effect considerably affecting the values of DTI metrics is the Gibbs ringing artifact^31,32^. Nowadays, this aspect is widely known. Since the Gibbs phenomenon always occurs due to the finite number of image encoding steps, more and more modern methods of its elimination are emerging, just like for denoising, including those based on machine learning^33^.

The effect of systematic errors on DTI metrics^14^ and tractography^24,34^ has been addressed and two major approaches for their elimination can be highlighted: gradient field correction using a coil tensor^25,28,35–37^ and the B matrix spatial distribution DTI calibration (BSD; the term BSD will also be used throughout the manuscript to denote an approach in which the B matrix used in the diffusion tensor calculations is spatially distributed as opposed to the standard approach, sDTI, which uses a single vendor-supplied B matrix for all voxels)^23,38^. Unlike the growing popularity of *b*-effective correction in medical spaces^39,40^, BSD corrects the entire B matrix voxel-by-voxel, which takes into account the spatial non-uniformity of a magnetic field gradient. Additionally, the BSD method is convenient because it corrects the total gradient occurring during the experiment including diffusion, imaging, eddy current-driven, background gradients and their cross-terms, during a single procedure. It is worth noting, that simple empirical methods are being sought to, for example, correct the nonlinearity of magnetic field gradients^25^.

In this study, we investigated the influence of denoising, Gibbs ringing removal and BSD calibration on DTI metrics and tractography. The analysis of DTI metrics was performed on an isotropic phantom. An anisotropy element for the additional analysis of diffusion tensor tractography was introduced through the study of an ex vivo rat brain. In addition to the element of microstructural influence, the ex vivo sample used was intended to eliminate the potential influence of motion, pulsation, induced gradients, and physiological noise, that can be problematic in the assessment of the processing pipeline^41^. Thanks to this, it was possible to study a purely physical effect that is inherent regardless of the object being examined and reduce the necessary preprocessing steps.

## Results

### Isotropic phantom study- whole volume analysis

**Figure 1:**
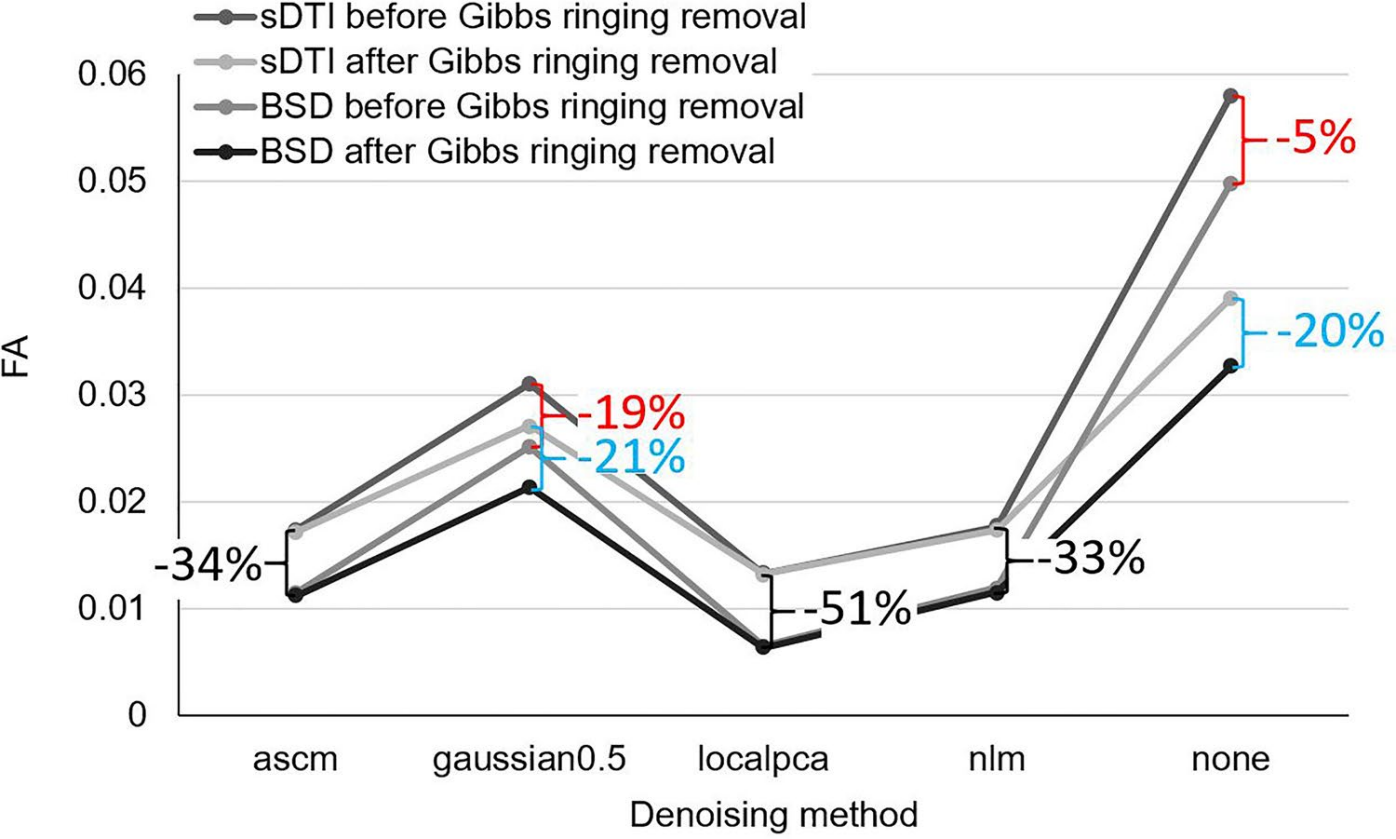
Fractional anisotropy, FA, values obtained from sDTI and BSD after the application of different denoising methods and before and after Gibbs ringing removal.

**Figure 2:**
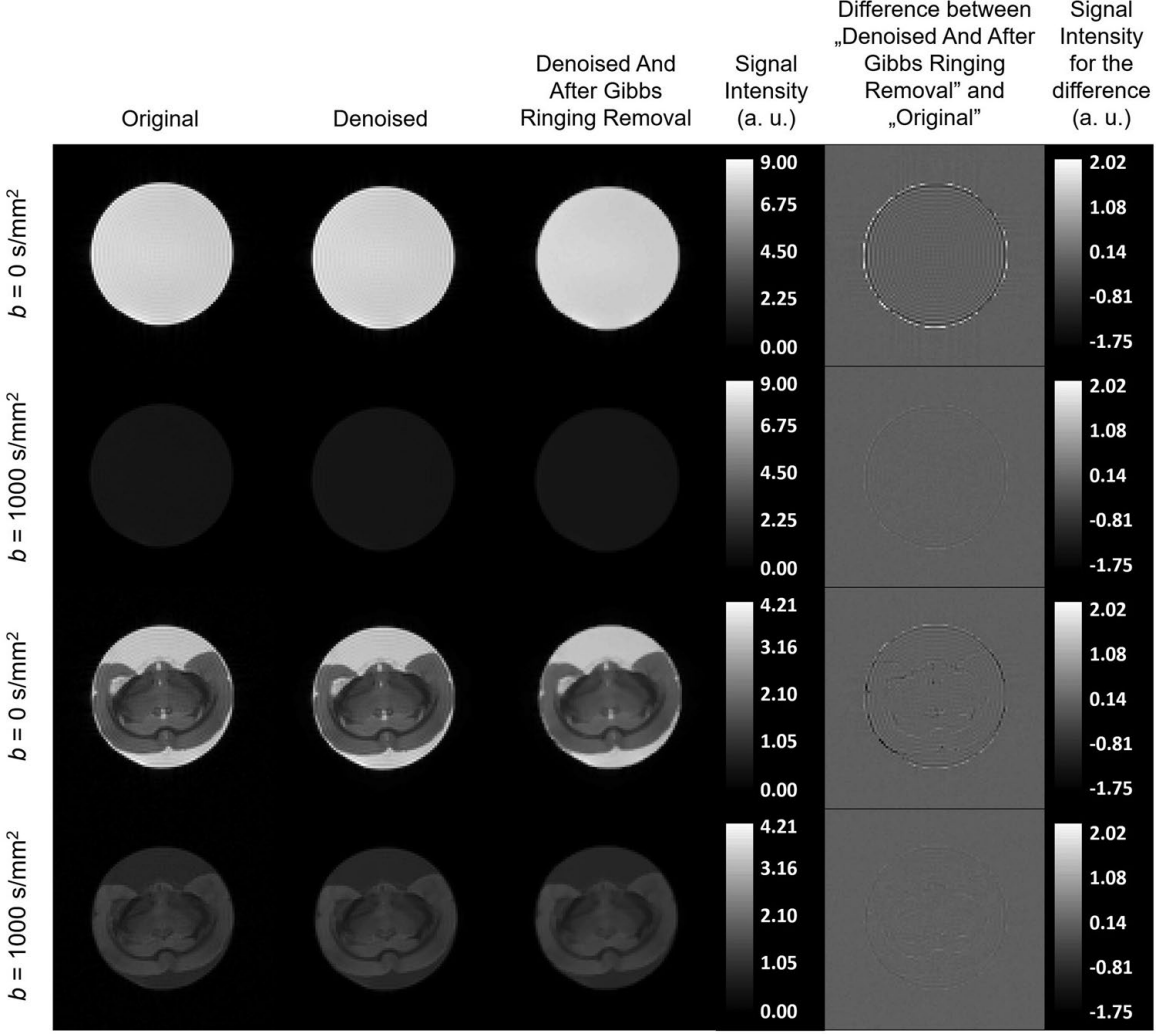
The DWI after denoising using local PCA and Gibbs ringing removal in isotropic phantom and rat brain in comparison to the original image for *b* = 0 s/mm^2^ and *b* = 1000 s/mm^2^ in an example (middle) slice. The last column presents the differential image of the original image and the one with the applied denoising and Gibbs ringing removal.

The performance of the denoising methods (adaptive soft coefficient matching method, blur using gaussian distribution function with standard deviation σ = 0.5, local principal component analysis (PCA), non-local means filter, marked as “ascm”, “gaussian0.5”, “localpca”, “nlm”, respectively, and “none” if no denoising was applied) was tested on the isotropic phantom. Denoising was conducted prior to Gibbs ringing removal (if applied). Then, FA values obtained from sDTI and BSD approaches were compared across denoising methods. The ground truth for isotropic phantom is FA = 0 and in case of diffusivities we assumed ${\lambda }_{1}={\lambda }_{2}={\lambda }_{3}=\text{MD}=1.976\pm 0.012\bullet {10}^{-3}$ mm^2^/s, which was determined from sDTI in a very small volume consisting of 207 voxels (equivalent to ~ 6.5 mm^3^) in the phantom’s isocenter. Those values should be obtained for the homogeneous isotropic phantom regardless the region of interest (ROI) size and location in space. Since FA is more sensitive to systematic errors than MD, the assessment of the denoising methods was based on FA values (Fig. 1). Local PCA showed the greatest performance by delivering the lowest FA independently of diffusion tensor calculation method (sDTI or BSD) and preprocessing. This method was chosen for the denoising in further research. It can also be seen that relative change of FA between sDTI and BSD was higher for stronger denoising. For any or weak denoising such as Gaussian blur with small σ, subsequent removal of the Gibbs ringing further improved the results noticeably. In the remaining methods, this effect was more subtle. The effect of denoising using local PCA and Gibbs ringing removal on the diffusion-weighted images (DWI) can be seen in Fig. 2.

### Isotropic phantom study- ROI-based approach

**Figure 3:**
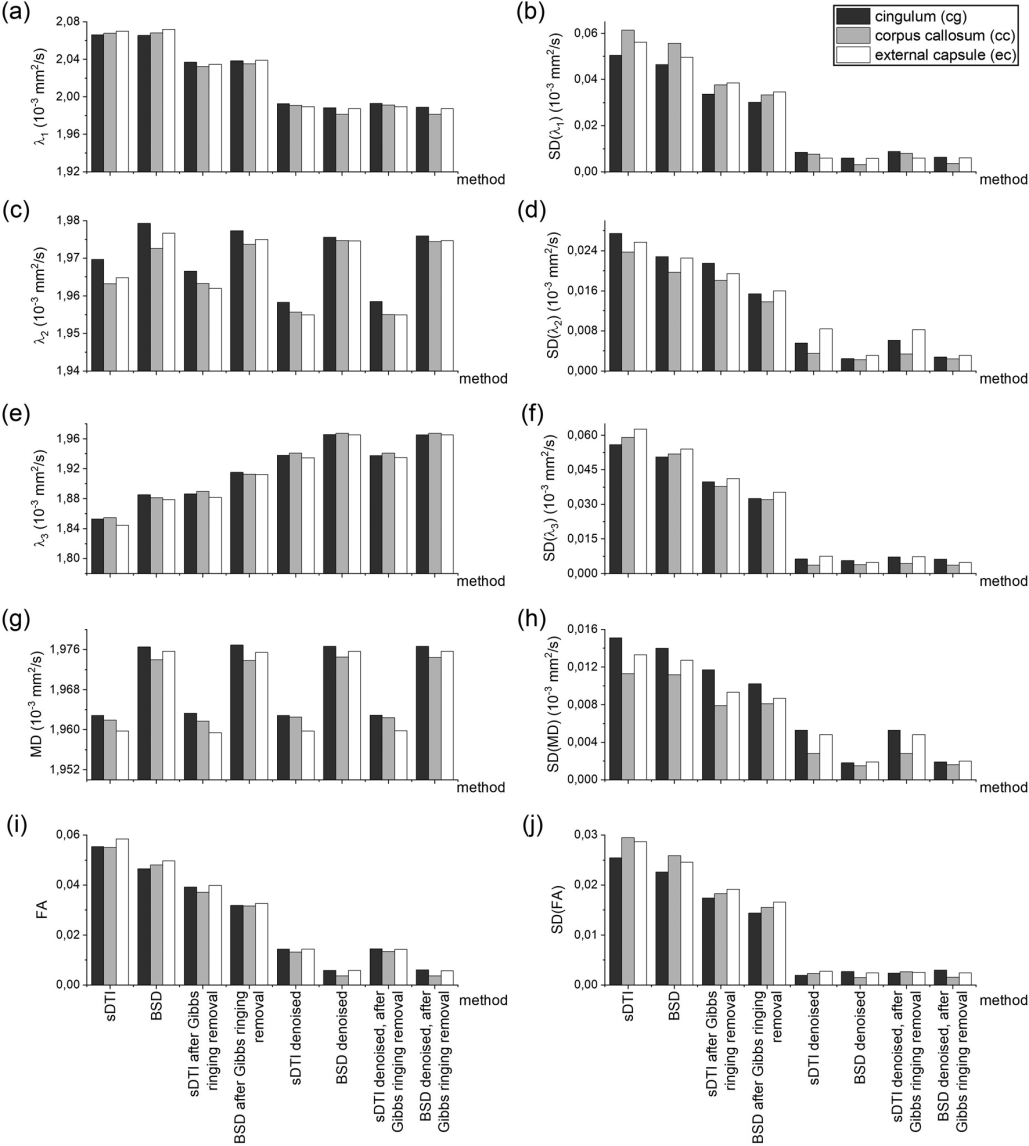
Standard diffusion tensor imaging (sDTI) and B matrix spatial distribution diffusion tensor imaging (BSD) metrics for the isotropic phantom in the regions representing rat brain ROIs (cg, cc, ec): first eigenvalue, λ_1_ (**a**), second eigenvalue, λ_2_ (**c**), third eigenvalue, λ_3_ (**e**), mean diffusivity, MD (**g**), fractional anisotropy, FA (**i**) and their standard deviations, SD (**b**, **d**, **f**, **h**, **j**, respectively). The x-axis representing methods is common for all graphs.

Magnetic field gradients non-uniformity and SNR inhomogeneity (peripherally to centrally)^15^ across the image can cause different systematic bias of DTI metrics depending on the ROI location. Therefore, in the next step, the sDTI and BSD metrics of the isotropic phantom in the regions representing rat brain ROIs (cg, cc, ec) were investigated for different preprocessing (Fig. 3, Supplementary Table S1). Based on Fig. 3 the following observations can be made:

- In general, the improvement of the metrics was observed in the following order of methods: BSD, sDTI after Gibbs ringing removal, BSD after Gibbs ringing removal, sDTI after denoising equally with sDTI after denoising and after Gibbs ringing removal, BSD after denoising equally with BSD after denoising and after Gibbs ringing removal;
- λ_1_ approached to the reference value after Gibbs ringing removal and even more after denoising with little difference between sDTI and BSD;
- λ_2_ approached the reference value after BSD calibration, while subsequent preprocessing steps deepened the underestimation in sDTI and evened out the differences among ROIs in BSD;
- λ_3_ approached the reference value with each step, while the value closest to the referential one was obtained for BSD after denoising or after denoising and after Gibbs ringing removal;
- MD reached the reference value only in BSD (1.9616∙10^–3^ mm^2^/s and 1.9755∙10^–3^ mm^2^/s in average for all sDTI- and BSD-based methods, respectively);
- FA improvement was lower and lower with each step (statistically significant differences with *p*-value < 0.001) approaching to the ground truth, reaching minimum for BSD after denoising or after denoising and after Gibbs ringing removal (0.0052 vs. 0.0141 for the sDTI with the same preprocessing, vs. 0.0562 for sDTI without preprocessing);
- Standard deviations of all metrics were improved with each step, with the greatest decrease after denoising for both sDTI and BSD.

### DTI metrics in the rat brain

**Figure 4:**
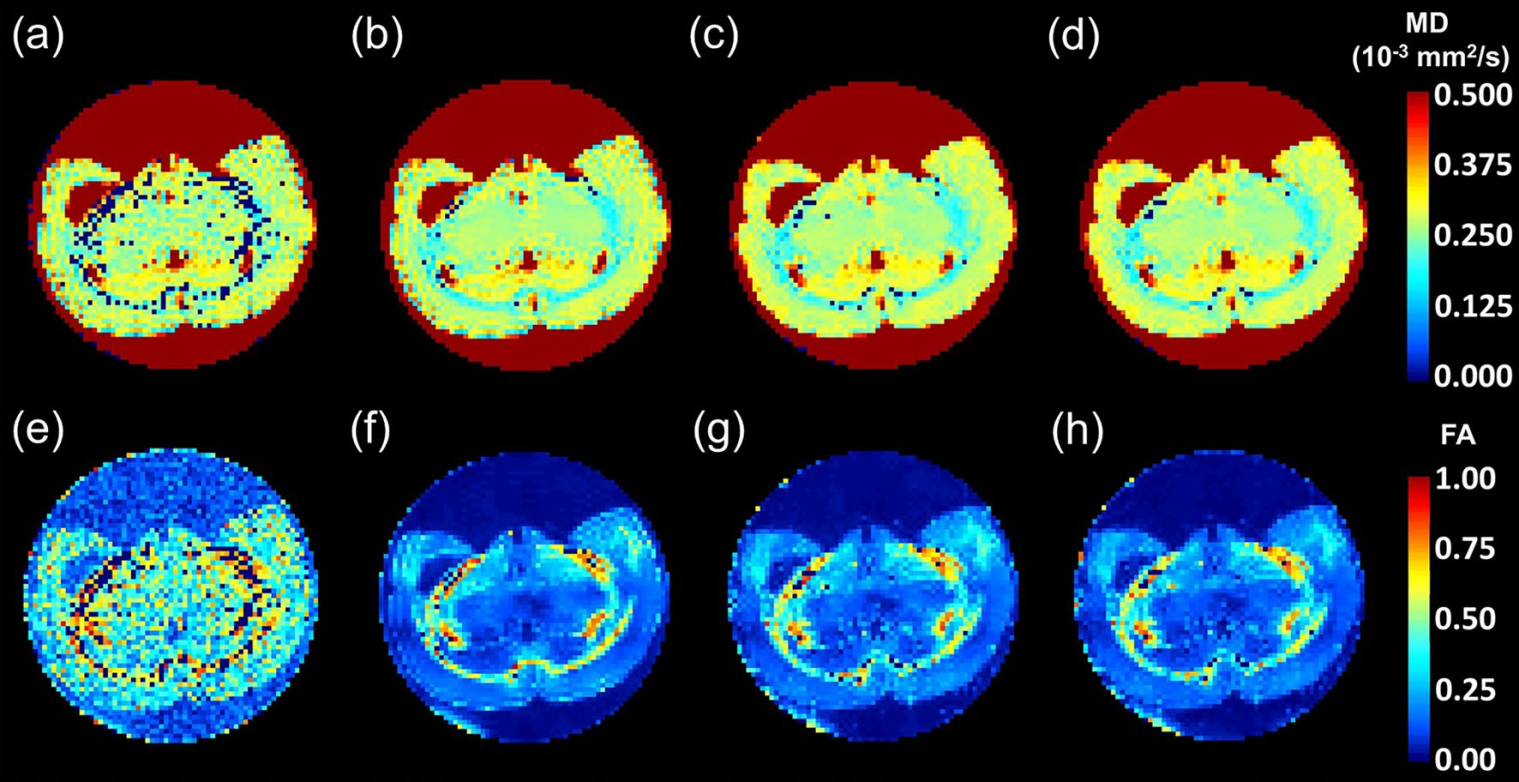
Mean diffusivity, MD (upper row) and fractional anisotropy, FA (bottom row) for the example (middle) slice obtained for sDTI (**a**,**e**), sDTI after Gibbs ringing removal (**b**,**f**), sDTI after denoising and after Gibbs ringing removal (**c**,**g**) and BSD after denoising and after Gibbs ringing removal (**d**,**h**).

The DWI obtained after denoising and Gibbs ringing removal applied to the rat brain are shown in Fig. 2. Example MD and FA maps are shown in Fig. 4. It can be seen that denoising substantially improved the quality of maps, while BSD calibration introduced local quantitative changes.

**Figure 5:**
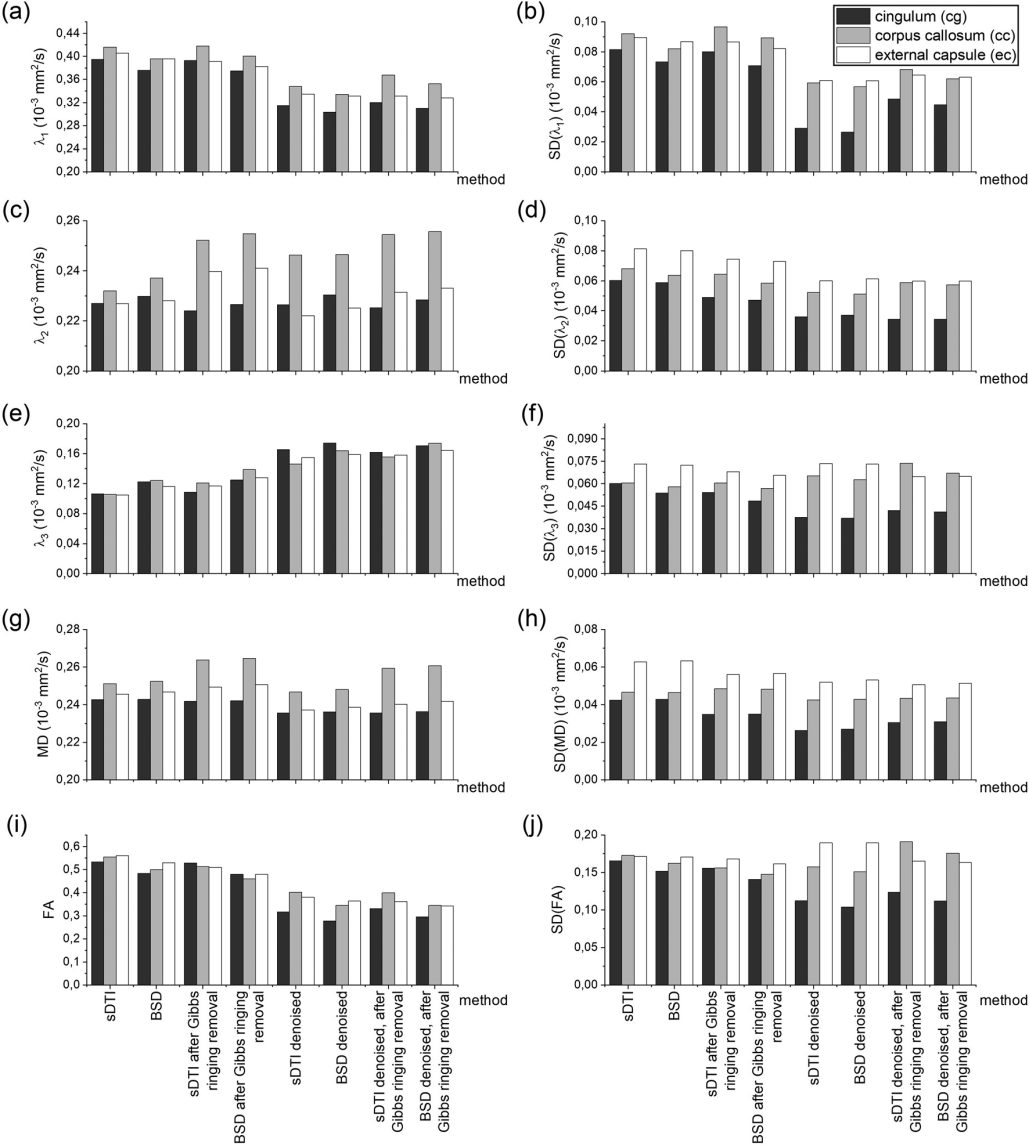
Standard diffusion tensor imaging (sDTI) and B matrix spatial distribution diffusion tensor imaging (BSD) metrics for the rat brain in three ROIs (cg, cc, ec): first eigenvalue, λ_1_ (**a**), second eigenvalue, λ_2_ (**c**), third eigenvalue, λ_3_ (**e**), mean diffusivity, MD (**g**), fractional anisotropy, FA (**i**) and their standard deviations, SD (**b**, **d**, **f**, **h**, **j**, respectively). The x-axis representing methods is common for all graphs.

In the case of the rat brain, the effect of preprocessing in sDTI and BSD was more subtle than in isotropic phantom (Fig. 5, Supplementary Table S2). FA value in each subsequent method (according to x-axis ticks in Fig. 5) was significantly different from the previous one in all ROIs (*p* value < 0.001). MD was significantly different between sDTI and BSD in cg and ec (*p* value < 0.001), while statistically insignificant differences between methods were found in cc (probably due to small number of pixels in the ROI and relatively high standard deviations that can result from anatomical differences).

### Rat brain tractography

**Figure 6:**
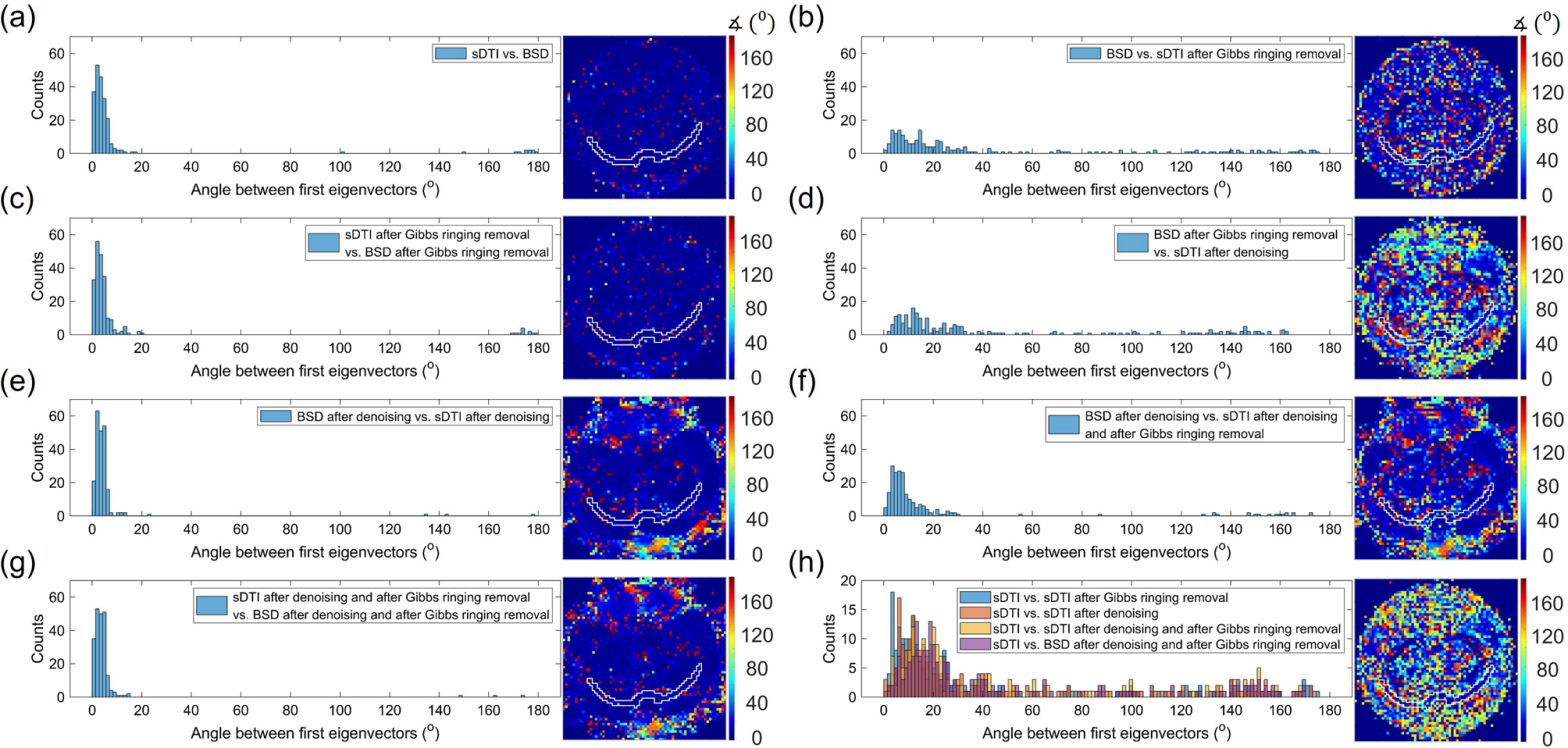
The histograms and associated maps of angles between the first eigenvectors coming from sDTI or BSD with different preprocessing as described in legends (**a**–**g**). Map in (**h**) was created based on sDTI and BSD after denoising and after Gibbs ringing removal.

Due to the nature of tractography algorithms, differences between tractograms may be imperceptible or completely masked despite differences in diffusion tensor metrics. When changing tractography parameters, these differences may become more pronounced. Since the key role in diffusion tensor tractography is played by the first eigenvector of the tensor, the first step in assessing differences between tractograms may be to analyze the distribution of differences in its direction in the ROI. Figure 6 shows the histograms of angle values between two first eigenvectors obtained with subsequent methods. It can be seen that if the same preprocessing is applied, the angle between the first eigenvector from sDTI and BSD had narrower distribution than in the case where sDTI was associated with different preprocessing than BSD. Moreover, the inclusion of the distributions of the first eigenvector direction differences between sDTI and BSD with different preprocessing (Fig. 6b,d,f) shows the effect at each subsequent point of the reduction of systematic errors (this order was determined based on FA in the isotropic phantom in Fig. 3). The distributions in Fig. 6a–g can be described by medians equal to 3.3, 18, 3.5, 27, 4.0, 9.2, 3.9 degrees and means equal to 9.1, 44, 13, 54, 9.4, 26, 9.9 degrees, respectively. In the last step, we checked the first eigenvector direction change after each subsequent step of the simplified pipeline (i.e. denoising → Gibbs ringing removal → BSD) that we proposed for our data, compared to the standard approach (sDTI without preprocessing). The distributions in Fig. 6h can be described by medians equal to 17, 25, 30, 31 degrees and means equal to 42, 50, 53, 56 degrees according to the order in the legend.

**Figure 7:**
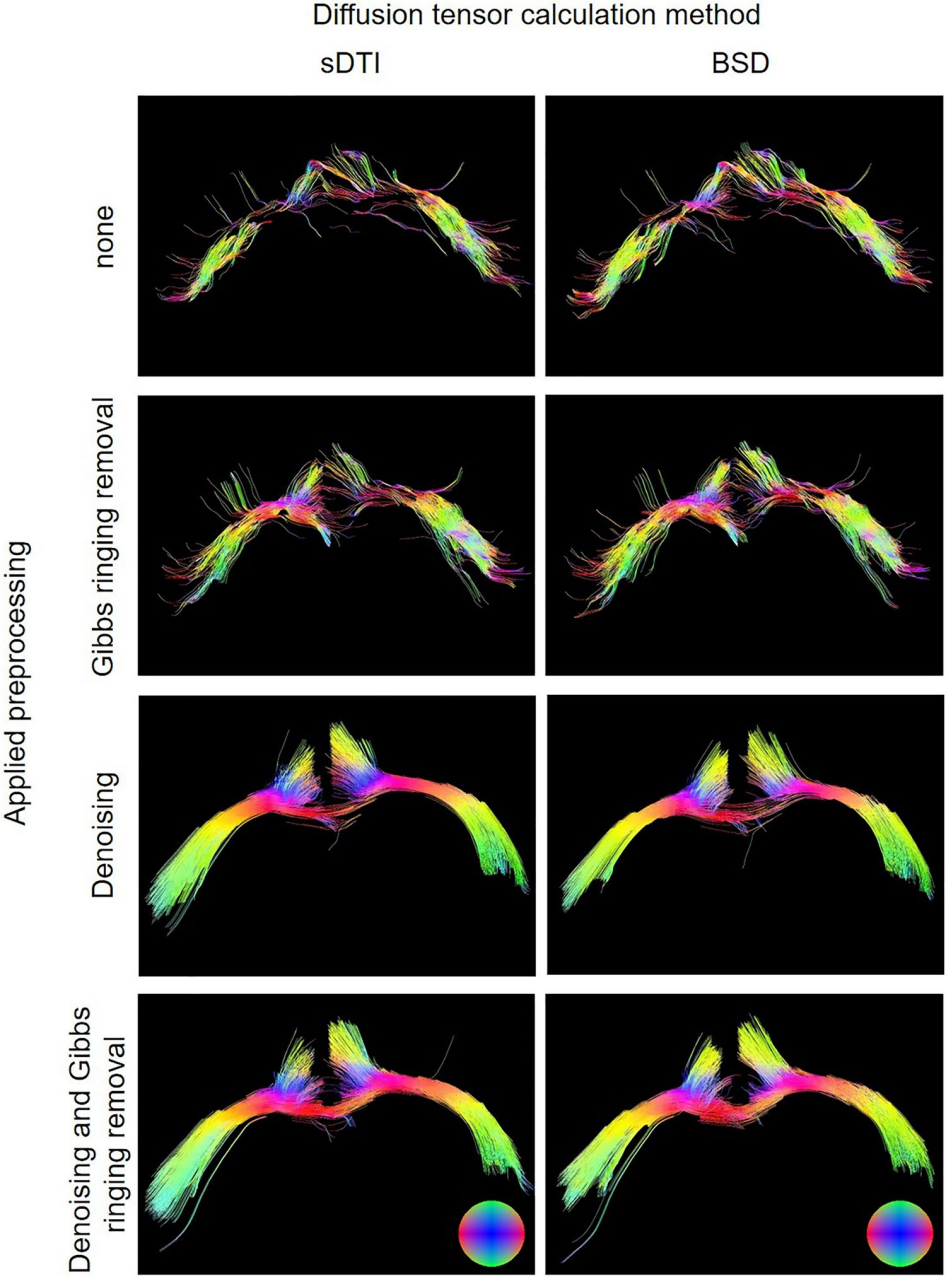
Diffusion tensor tractography obtained for sDTI (left column) and BSD (right column) after various preprocessing: none, denoising, Gibbs ringing removal and denoising and Gibbs ringing removal. Color balls indicate the frame directions.

In the last step, the fiber tracking obtained from sDTI and BSD after different preprocessing was compared. Large differences across the methods are visible (Fig. 7). The key step was denoising, after which density, anatomical correctness and tract shape (smooth tracts without protrusive fibers) were visibly improved (Fig. 7). Quantitative tractography indices showed an increase in fiber density relative to sDTI depending on the correction method (Table 1). The first noticeable increase was observed for BSD, then for sDTI/BSD after removing Gibbs ringing, then for sDTI/BSD after denoising, and the largest for sDTI/BSD after denoising and after removing Gibbs ringing. The outperformance of BSD over sDTI with respect to fiber density decreased with the number of preprocessing steps. Table 1 also shows mean projections of the tracts on each axis in ROIs. They allowed us to notice that these subtle local differences in the tractography obtained from sDTI and BSD also caused noticeable differences globally (within the entire ROI).

**Table 1:** Density (in number of tracts per total number of pixels in a ROI) and the length of the unit vector representing the average direction of fiber tract bundle in each dimension of the system (x, y and z, designated as projections X, Y, Z).

Method	ROI	Density		The length of the first eigenvector					
				Projection X		Projection Y		Projection Z	
		Mean	Sd	Mean	Sd	Mean	Sd	Mean	Sd
sDTI	cg	3.96	4.18	0.31	0.25	0.27	0.28	0.48	0.38
BSD		6.70	6.25	0.38	0.27	0.31	0.27	0.51	0.34
sDTI after Gibbs ringing removal		10.09	12.25	0.50	0.29	0.22	0.21	0.51	0.33
BSD after Gibbs ringing removal		13.81	13.15	0.53	0.23	0.31	0.25	0.58	0.28
sDTI after denoising		15.95	11.60	0.45	0.27	0.15	0.07	0.78	0.23
BSD after denoising		14.74	9.83	0.49	0.22	0.22	0.14	0.75	0.21
sDTI after denoising, after Gibbs ringing removal		18.67	11.38	0.54	0.21	0.27	0.19	0.70	0.22
BSD after denoising, after Gibbs ringing removal		17.56	12.44	0.57	0.18	0.30	0.20	0.64	0.25
sDTI	cc	0	0	0	0	0	0	0	0
BSD		0.73	1.21	0.21	0.37	0.06	0.14	0.10	0.24
sDTI after Gibbs ringing removal		1.64	2.29	0.45	0.40	0.23	0.24	0.20	0.21
BSD after Gibbs ringing removal		2.52	3.19	0.51	0.37	0.29	0.26	0.22	0.22
sDTI after denoising		3.09	3.60	0.54	0.42	0.13	0.14	0.22	0.27
BSD after denoising		3.97	3.43	0.76	0.34	0.20	0.16	0.23	0.19
sDTI after denoising, after Gibbs ringing removal		12.00	8.40	0.94	0.05	0.23	0.13	0.17	0.12
BSD after denoising, after Gibbs ringing removal		13.85	8.09	0.88	0.17	0.25	0.15	0.21	0.13
sDTI	ec	4.15	6.57	0.38	0.35	0.32	0.33	0.18	0.23
BSD		7.26	10.20	0.48	0.34	0.38	0.33	0.21	0.22
sDTI after Gibbs ringing removal		8.69	9.93	0.64	0.29	0.44	0.29	0.22	0.18
BSD after Gibbs ringing removal		11.12	10.11	0.69	0.24	0.47	0.27	0.23	0.17
sDTI after denoising		24.91	16.59	0.70	0.25	0.45	0.30	0.24	0.18
BSD after denoising		20.49	13.55	0.70	0.23	0.48	0.29	0.26	0.17
sDTI after denoising, after Gibbs ringing removal		27.20	15.60	0.73	0.23	0.46	0.29	0.24	0.16
BSD after denoising, after Gibbs ringing removal		27.40	17.94	0.72	0.21	0.48	0.28	0.25	0.16
sDTI	All	3.80	6.12	0.34	0.34	0.29	0.32	0.21	0.27
BSD		6.67	9.50	0.45	0.34	0.35	0.32	0.24	0.26
sDTI after Gibbs ringing removal		8.33	10.12	0.61	0.31	0.39	0.29	0.26	0.23
BSD after Gibbs ringing removal		10.81	10.55	0.65	0.26	0.43	0.28	0.28	0.23
sDTI after denoising		21.98	16.59	0.66	0.28	0.38	0.30	0.31	0.27
BSD after denoising		18.41	13.41	0.68	0.25	0.42	0.29	0.32	0.25
sDTI after denoising, after Gibbs ringing removal		24.85	15.39	0.72	0.23	0.42	0.28	0.29	0.23
BSD after denoising, after Gibbs ringing removal		25.00	17.34	0.71	0.22	0.44	0.28	0.30	0.22

## Discussion

This study aimed to detect systematic errors in high-resolution DTI and evaluate a proposed correction pipeline to eliminate them. Choosing a DTI preprocessing pipeline is complex due to factors like cost, time, and lack of standardization^42,43^. The BSD method was introduced to address errors from non-uniform magnetic field gradients. Various and only necessary preprocessing methods combined with BSD were tested for optimal systematic bias removal. Using an isotropic phantom provided ground truth for data comparison, and a fixed post-mortem rat brain helped eliminate movement-related artifacts. Despite some disadvantages, this sample reduced the need for certain preprocessing steps. Results showed BSD outperformed the standard sDTI approach. The study found denoising significantly improved metrics and tractography, with additional benefits from removing Gibbs ringing and applying BSD. BSD showed higher performance after denoising and Gibbs ringing removal, effectively recognizing magnetic field gradient patterns. Mean diffusivity bias was nearly eliminated with BSD, a result not achieved by the standard sDTI method.

Analysis of the results from the isotropic phantom in its entire volume showed that the performance of the BSD method is influenced by the denoising method and whether Gibbs ringing removal was used. When averaging the metrics over the entire phantom’s volume, the closest to the reference FA values were the metrics from BSD after denoising and after Gibbs ringing removal.

ROI-based isotropic phantom analysis was intended to investigate the degree of bias in specific areas where the rat brain was analyzed. This is a special type of analysis because ROIs have different spatial locations and sizes. As is known, both gradient inhomogeneity and SNR vary depending on the location in space^15,25^. The size of the ROI also determines the amount of bias in the diffusion tensor metrics, because for a smaller ROI the mean can be more systematically shifted^11^. The results of the ROI-based analysis showed that the metrics from BSD after denoising and BSD after denoising and after Gibbs ringing removal were closest to the reference values. However, it is worth noting that in some cases removing Gibbs ringing after denoising introduced additional bias rather than removing it. This was the case, for example, in the cc ROI. Here it should be taken into account that the value of the metric cannot be the only indicator of the correction performance, but it should be associated with the image quality assessment.

The SNR in the rat brain obtained for *b* = 0 and *b* = 1000 s/mm^2^ was equal to 41/34 (32/31 dB) and 51/41 (34/32 dB) in cc + ec and cg, respectively. Despite that it is claimed that for *b* = 1000 s/mm^2^ an SNR > 10 (~ 30 dB) is required for the accurate determination of DTI metrics (^9^ and ref. therein), our results showed that denoising played a significant role in their improving with respect to the sDTI. For example, the relative change of FA in sDTI after denoising with respect to the sDTI was equal to − 41%, − 28% and − 32% in cg, cc and ec, respectively. The application of BSD introduced the additional change of ~ − 5–− 14% depending on ROI. Reducing FA is equated in the literature with removing systematic errors^14,18,19^. By correcting all elements of the B matrix voxel-by-voxel, all elements of the diffusion tensor matrix are corrected differently in these voxels. This also affects the solutions of the eigenequation of the diffusion tensor, including the directions of the first eigenvector. Again, it can be seen that image denoising had the greatest impact on its direction (Fig. 6). However, changes in the direction of the first eigenvector were observed between every two consecutive steps ranging from 0 to 180 degrees. The use of the BSD method with recpect to sDTI for the data after denoising and after Gibbs ringing removal resulted in a median angle of 10 degrees inside ROIs compared to the standard method. Despite the small local difference, such small voxel-by-voxel changes can result in a completely different tractography.

Diffusion tensor tractography showed an incomparable visual change for the data after denoising (Fig. 7). Some local differences are also visible after subsequent Gibbs ringing removal and BSD application, for example left cingulum and corpus callosum. Quantitative analysis of the average fiber densities and directions in the ROI showed that a difference in tractographic fiber directions and densities can not only be found locally but also globally. Considering that this is the average of all fiber directions from the bundle averaged first within the voxel and then across the entire ROI, differences between sDTI and BSD can still be seen (in means or standard deviations) (Table 1).

Unfortunately, there is no ground truth for rat DTI metrics and comparisons are made relative to sDTI. However, the design of the study allows for certain assumptions. First, an isotropic phantom was investigated, for which theoretical FA and MD can be determined. Based on the results from the phantom it is possible to (a) detect and investigate the effects of systematic errors and (b) compare the performance of different preprocessing and choose the optimal pipeline. Then, it can be assumed that similar effects are observed in the rat brain. In the case of statistical error, we can expect that it is increased in the rat brain due to lower diffusivity and anisotropy of the system. Therefore, the same methods applied to the isotropic phantom and the brain can evince different performance in removing systematic bias. However, it can be assumed that ex vivo sample have the same distribution of magnetic field gradients and that the pipeline that worked the best for the phantom is also of choice for the rat brain. Since the performance of BSD method and denoising are dependent, one can at most assume that the effectiveness of systematic bias removal would not be as accurate as in the phantom case.

BSD was proposed as the final step in correcting DTI data after preprocessing. It has many advantages over mathematical methods. The BSD method not only allows the calibration of non-uniform diffusion gradients, but it also corrects systematic errors related to the total magnetic field gradient occurring during the time from excitation to echo registration in one procedure. In addition to diffusion gradients, it includes contributions from imaging gradients, background gradients and others, for example those resulting from eddy currents, and their cross-terms. This makes it possible to reduce pre-processing procedures. Its effectiveness was confirmed theoretically^44,45^, in simulations^14,24^, in clinical measurements in a medium magnetic field^46^ and on an anisotropic phantom in a high magnetic field^23^. In this study, its performance and usefulness in DTI was tested on an anatomical system characterized by low diffusivity, for high spatial resolution and in the presence of Gibbs ringing artifact. This is an important step because, as it transpired, BSD applied to the original data cannot fully improve the DTI metrics (Fig. 1). Moreover, the applicability of BSD was never investigated in such high spatial resolution. In the study we observed that along noise, magnetic field gradient imperfections can have already impact in the voxel size of 250 × 250 × 500 μm. This should be kept in mind, because it is often assumed that magnetic field gradients are uniform in the small volume at the magnet isocenter. The analysis showed that the use of pre-processed data is important for improving DTI metrics and tractography in the standard method, sDTI, but also for improving the performance of the BSD method. This is most likely related to the high image resolution and therefore, low SNR in the voxel. It can be seen that the BSD performance in the isotropic phantom on the same scanner for an almost tenfold lower in-plane resolution was much higher without denoising (even a 14-fold decrease in FA)^23^. However, in the capillary phantom (capillary inner diameter 30 μm), it caused only a -2% change in FA compared to sDTI.

In the study, denoising was conducted by using local PCA method. For this method we obtained the FA value closest to the theoretical FA = 0 in isotropic phantom and the lowest standard deviation across the whole volume (not shown). For comparison, we checked the performance of MPPCA method commonly used in preprocessing pipelines based on isotropic phantom DTI. Unfortunately, the FA obtained for BSD after MPPCA was 1.9–2.8 times higher (FA≈0.01 versus FA≈0.006 after MPPCA and local PCA, respectively), while the standard deviation was 2.2–5.7 times higher than for local PCA depending on ROI. Results were slightly improved after Gibbs ringing removal. This is in accordance with another study, confirming the outperformance of local PCA over MPPCA^47^. Results also suggest that weak or non-aggressive denoising might be insufficient for high resolution DTI.

To sum up, the use of the BSD method to remove systematic errors in DTI metrics and tractography is an alternative to mathematical methods. The entire side effect associated with imperfect magnetic field gradients can be corrected in a single procedure. Full correction of the bias of the metrics and diffusion tensor tractography required the use of preprocessing. The developed pipeline consisted of local PCA denoising, Gibbs ringing removal and the application of the BSD method. In this configuration, BSD outperformed sDTI in removing the bias of diffusion tensor metrics relative to reference values. In some cases, removing Gibbs ringing caused worsening of BSD performance, resulting in poorer elimination of the systematic bias than BSD with denoising alone. However, it seems that the artifact identified in the image should be corrected at the expense of a slightly worse DTI metrics result. The next steps in BSD research should incorporate in vivo elements intentionally eliminated in this work. By making the measurements independent of object movement and physiological noise, it was possible to investigate purely physical effects that are undesirable in DTI. This is important because they are independent of the tested object but uniquely dependent on the scanner and sequence. This means that they always appear in the obtained results, and for in vivo studies they may be an additional source of errors in quantitative analysis.

## Methods

### Sample preparation

In the study one isotropic, glass ball water phantom and one rat brain were examined. The isotropic phantom had a bulk self-diffusion coefficient, *D*_*0*_, equal to *D*_*0*_ = 1.976∙10^–3^ mm^2^/s (determined in a volume of 6.5 mm^3^ in the phantom and magnet isocenter) and fractional anisotropy, FA = 0. Those values were considered the ground truth in the assessment of the performance of the preprocessing and calibration methods. The study also involved a single, adult, healthy, male, 6 month old Wistar rat. The animal was given a lethal dose of pentobarbital, and perfused transcardially with 0.9% NaCl (Polpharma, Poland) followed by 10% formalin in 0.1 M phosphate buffer (pH 7.4, Chempur, Poland). Then, the brain was removed from the skull and postfixed in formalin. No procedures were performed before the animal was sacrificed. The study is reported in accordance with ARRIVE guidelines. All experiments were performed in accordance with relevant guidelines and regulations and were approved by the. All activities connected to the breeding conditions, killing and brain extraction were in accordance with the Directive 2010/63/EU of the European Parliament and of the Council of 22 September 2010 on the protection of animals used for scientific purposes and the national law, were approved by the Animal Care and Use Committee of the Jagiellonian University in Krakow. DTI examination was conducted at the Henryk Niewodniczański Institute of Nuclear Physics PAN in Krakow using standard protocol.

### Experiments

The DTI of the isotropic phantom and the rat brain was conducted in The Institute of Nuclear Physics, Polish Academy of Sciences in Krakow, on a 9.4 T Bruker Biospec 94/20 (Billerica, MA, USA) using a standard pulsed gradient spin echo echo planar imaging pulse sequence in the axial plane and standard protocol. The DTI sequence parameters were as follows: 6 diffusion gradient directions; voxel size of 250 × 250 × 500 μm; diffusion gradient pulse duration, δ = 5 ms, diffusion time, Δ = 8.52 ms; effective *b*-values, *b* = 26 (to which we refer as *b* = 0 s/mm^2^) and *b* = 940–997 s/mm^2^ (to which we refer as *b* = 1000 s/mm^2^); repetition time, *TR* = 10 s, number of averages, *NoA* = 4; echo time, *TE* = 20 ms. The total time of the experiment was equal to 9 h 57 min and 20 s.

### Image preprocessing

Preprocessing was carried out in Python DIPY software^48^ and encompassed denoising and next, Gibbs ringing removal^49,50^. Four methods of denoising were tested on the isotropic phantom: Non-local means filter^51,52^ (nlm), Adaptive Soft Coefficient Matching^51^ (ascm), Local principal component analysis (PCA) via empirical thresholds^53^ (localpca), which were implemented in DIPY and Gaussian blurring filter with a radius of a neighborhood equal to 10 pixels and standard deviation of the distribution σ = 0.5 that was performed using the “BSD” in-house software (ver. 2.0, AGH, Krakow). Gaussian blur was obtained with a standard approach using Gaussian function in 2D $f\left(x,y\right)=\frac{1}{2\pi {\sigma }^{2}}\text{exp}\left(-\frac{{x}^{2}+{y}^{2}}{2{\sigma }^{2}}\right)$, where x and y are coordinations of the voxel in the image plane and standard deviation, σ, is scale parameter. The best filter was chosen based on the DTI metrics obtained for the isotropic phantom with known diffusional properties (*D*_*0*_ = 1.976∙10^–3^ mm^2^/s and FA = 0). For comparison, commonly used Marchenko-Pastur PCA (MPPCA) denoising method^54,55^ was applied to isotropic phantom by using MRtrix3 software^8^.

### Diffusion tensor calculation

In the study, two approaches for diffusion tensor calculation were applied. The first one was the standard approach using constant in space B matrix provided by the vendor (sDTI). The second one used spatially distributed B matrix for each of the diffusion gradient direction (BSD). BSD constitutes the last type of correction- the calibration of imperfect (non-uniform or nonlinear) magnetic field gradients using the B matrix spatial distribution (BSD) method^8^. BSD detects and corrects for systematic errors according to the general rules described in detail theoretically and experimentally^23,44^. In general, this is a method involving the use of appropriately determined b matrices voxel-by-voxel, b(r), for each DWI. All the technical assumptions of the method are also described in detail in the patent application published^8^. In practice, seven b(r), i.e. for *b* = 0 s/mm^2^ image and each DWI, were determined from a phantom’s twin DTI measurements (i.e. with exactly the same protocol as the target object) as described in the example clinical application^46^.

The two approaches were applied to: (a) original data; (b) data after Gibbs ringing removal; (c) data after denoising and (d) data after denoising and after Gibbs ringing removal (where preprocessing steps were applied in this order). Hence, in the study the total of eight diffusion tensors were determined and named with respect to the procedures that we performed on the data: sDTI, sDTI after Gibbs ringing removal, sDTI after denoising, sDTI after denoising and after Gibbs ringing removal, BSD, BSD after Gibbs ringing removal, BSD after denoising, BSD after denoising and after Gibbs ringing removal.

To the best of our knowledge, the calculation approach using BSD is not available in the usual DTI software and hence, all diffusion tensor calculations (sDTI and BSD) were carried out in our “BSD” software (a part of the software utility is available as an online tool^56^).

### Diffusion tensor analysis

The denoising method was chosen based on the lowest value of FA in the isotropic phantom calculated in the big ROI encompassing the whole volume of the phantom (excluding edges). ROI-based analysis was conducted in three manually determined regions of the rat brain: cingulum (cg), corpus callosum (cc) and external capsule (ec), in isotropic phantom as well as in the rat brain. The angles between the two eigenvectors coming from two diffusion tensors were calculated, analyzed and visualized in MATLAB software (The MathWorks Inc., MATLAB Version: 23.2.0.2409890 (R2023b) Update 3, Natick, Massachusetts, USA).

### Diffusion tensor tractography

The diffusion tensor tractography calculation and visualization was conducted in the “BSD” software using a simple streamlined algorithm with the following parameters: FA threshold = 0.14, angular threshold = 45°, step = 0.1, seed step = 0.1, minimal fiber length = 20. The software also enables getting information about the mean density and resultant vector representing the average direction of a fiber tract bundle inside a voxel. Fibertracking quantities analyzed in the study can be defined as follows:

- Density—number of fiber tracts per voxel (in a given ROI it is summed over the voxels and divided by their number),
- Projection X/Y/Z- orthogonal projection on the X/Y/Z axis of the resultant unit vector, which is the vector sum of the directions of all tractographic fibers in the voxel (in a given ROI it is summed over the voxels and divided by their number).

### Statistical analysis

Applied methods were ordered according to the preprocessing steps as listed in section “Diffusion tensor calculation”. Next, statistically significant differences were evaluated between two subsequent methods as well as between each method and the standard approach, sDTI. The Wilcoxon signed rank test was used at the significance level of 0.05 and then, false discovery rate (FDR) correction was applied for each ROI separately based on the method introduced by Storey^57^. The Wilcoxon signed rank test requires vector data, which in our case consisted of voxel-by-voxel FA or MD values. Statistical analysis was performed in MATLAB software (The MathWorks Inc., MATLAB Version: 23.2.0.2409890 (R2023b) Update 3, Natick, Massachusetts, USA).

## Data Availability

The datasets generated and analyzed during the current study are available from the corresponding author on reasonable request.

## Supplementary Tables

Supplementary Tables.
