# Supplementary material for: The effect of elimination of gibbs ringing, noise and systematic errors on the DTI metrics and tractography in a rat brain
Source: Sci Rep. 2024 Jul 1;14:15010. doi: 10.1038/s41598-024-66076-z (PMC11217413; doi:10.1038/s41598-024-66076-z)
Supplement: Supplementary file 1 — Supplementary Tables. [file 41598_2024_66076_MOESM1_ESM.pdf]

# SUPPLEMENTARY INFORMATION

# The Effect of Elimination of Gibbs Ringing, Noise and Systematic Errors on the DTI Metrics and Tractography in a Rat Brain

*Weronika Mazur-Rosmus[1], Artur T. Krzyżak[1,*]*

[1]AGH University of Krakow, al. Mickiewicza 30, 30-059 Krakow, Poland

*Corresponding author: akrzyzak@agh.edu.pl

**Supplementary Table S1.** Diffusion tensor metrics obtained in the isotropic phantom in regions spatially representing cingulum (cg), corpus callosum (cc) and external capsule (ec) of a rat brain from diffusion tensors calculated using different methods and preprocessing. $\lambda_{1,2,3}$ are three eigenvalues ($10^{-3}$ $mm^2/s$), MD is mean diffusivity ($10^{-3}$ $mm^2/s$), FA is fractional anisotropy.

| ROI | method | Mean value | | | | | Standard deviation | | | | |
|---|---|---|---|---|---|---|---|---|---|---|---|
| | | $\lambda_1$ | $\lambda_2$ | $\lambda_3$ | MD | FA | $\lambda_1$ | $\lambda_2$ | $\lambda_3$ | MD | FA |
| cg | sDTI | 2.0661 | 1.9697 | 1.8526 | 1.9628 | 0.0553 | 0.0505 | 0.0274 | 0.0560 | 0.0151 | 0.0254 |
| | BSD | 2.0653 | 1.9793 | 1.8850 | 1.9765 | 0.0465 | 0.0465 | 0.0228 | 0.0505 | 0.0140 | 0.0226 |
| | sDTI after Gibbs ringing removal | 2.0370 | 1.9666 | 1.8862 | 1.9633 | 0.0392 | 0.0337 | 0.0215 | 0.0398 | 0.0117 | 0.0174 |
| | BSD after Gibbs ringing removal | 2.0384 | 1.9773 | 1.9150 | 1.9769 | 0.0318 | 0.0301 | 0.0154 | 0.0326 | 0.0102 | 0.0144 |
| | sDTI denoised | 1.9926 | 1.9583 | 1.9375 | 1.9628 | 0.0143 | 0.0085 | 0.0056 | 0.0063 | 0.0053 | 0.0019 |
| | BSD denoised | 1.9883 | 1.9756 | 1.9657 | 1.9766 | 0.0058 | 0.0060 | 0.0025 | 0.0056 | 0.0018 | 0.0027 |
| | sDTI denoised, after Gibbs ringing removal | 1.9929 | 1.9585 | 1.9373 | 1.9629 | 0.0145 | 0.0088 | 0.0061 | 0.0072 | 0.0053 | 0.0024 |
| | BSD denoised, after Gibbs ringing removal | 1.9888 | 1.9759 | 1.9652 | 1.9766 | 0.0061 | 0.0063 | 0.0028 | 0.0062 | 0.0019 | 0.0030 |
| cc | sDTI | 2.0679 | 1.9632 | 1.8546 | 1.9619 | 0.0550 | 0.0613 | 0.0237 | 0.0591 | 0.0113 | 0.0295 |
| | BSD | 2.0682 | 1.9726 | 1.8812 | 1.9740 | 0.0480 | 0.0557 | 0.0197 | 0.0519 | 0.0112 | 0.0259 |
| | sDTI after Gibbs ringing removal | 2.0323 | 1.9633 | 1.8896 | 1.9617 | 0.0371 | 0.0376 | 0.0181 | 0.0377 | 0.0079 | 0.0183 |
| | BSD after Gibbs ringing removal | 2.0352 | 1.9737 | 1.9125 | 1.9738 | 0.0316 | 0.0333 | 0.0138 | 0.0321 | 0.0081 | 0.0155 |
| | sDTI denoised | 1.9910 | 1.9556 | 1.9410 | 1.9625 | 0.0132 | 0.0076 | 0.0035 | 0.0035 | 0.0028 | 0.0024 |
| | BSD denoised | 1.9815 | 1.9747 | 1.9673 | 1.9745 | 0.0037 | 0.0031 | 0.0022 | 0.0039 | 0.0015 | 0.0015 |
| | sDTI denoised, after Gibbs ringing removal | 1.9914 | 1.9550 | 1.9407 | 1.9624 | 0.0134 | 0.0080 | 0.0034 | 0.0044 | 0.0028 | 0.0026 |
| | BSD denoised, after Gibbs ringing removal | 1.9815 | 1.9744 | 1.9672 | 1.9744 | 0.0037 | 0.0036 | 0.0024 | 0.0036 | 0.0016 | 0.0016 |
| ec | sDTI | 2.0699 | 1.9648 | 1.8446 | 1.9597 | 0.0584 | 0.0562 | 0.0257 | 0.0627 | 0.0133 | 0.0287 |
| | BSD | 2.0717 | 1.9767 | 1.8787 | 1.9757 | 0.0496 | 0.0497 | 0.0225 | 0.0540 | 0.0127 | 0.0246 |
| | sDTI after Gibbs ringing removal | 2.0347 | 1.9619 | 1.8816 | 1.9594 | 0.0398 | 0.0385 | 0.0194 | 0.0411 | 0.0093 | 0.0192 |

| | | | | | | | | | | | |
|---|---|---|---|---|---|---|---|---|---|---|---|
| | BSD after Gibbs ringing removal | 2.0388 | 1.9750 | 1.9124 | 1.9754 | 0.0326 | 0.0346 | 0.0160 | 0.0353 | 0.0087 | 0.0166 |
| | sDTI denoised | 1.9896 | 1.9549 | 1.9345 | 1.9597 | 0.0144 | 0.0060 | 0.0084 | 0.0076 | 0.0048 | 0.0028 |
| | BSD denoised | 1.9873 | 1.9746 | 1.9651 | 1.9757 | 0.0058 | 0.0058 | 0.0031 | 0.0048 | 0.0019 | 0.0024 |
| | sDTI denoised, after Gibbs ringing removal | 1.9895 | 1.9549 | 1.9348 | 1.9598 | 0.0143 | 0.0060 | 0.0082 | 0.0073 | 0.0048 | 0.0026 |
| | BSD denoised, after Gibbs ringing removal | 1.9872 | 1.9747 | 1.9653 | 1.9757 | 0.0057 | 0.0061 | 0.0031 | 0.0048 | 0.0020 | 0.0025 |

**Supplementary Table S2.** Diffusion tensor metrics obtained in cingulum (cg), corpus callosum (cc) and external capsule (ec) of a rat brain from diffusion tensors calculated using different methods and preprocessing. $\lambda_{1,2,3}$ are three eigenvalues ($10^{-3}$ $mm^2/s$), MD is mean diffusivity ($10^{-3}$ $mm^2/s$), FA is fractional anisotropy.

| ROI | method | Mean value | | | | | Standard deviation | | | | |
|---|---|---|---|---|---|---|---|---|---|---|---|
| | | $\lambda_1$ | $\lambda_2$ | $\lambda_3$ | MD | FA | $\lambda_1$ | $\lambda_2$ | $\lambda_3$ | MD | FA |
| cg | sDTI | 0.394 | 0.227 | 0.106 | 0.243 | 0.533 | 0.081 | 0.060 | 0.060 | 0.042 | 0.166 |
| | BSD | 0.376 | 0.230 | 0.122 | 0.243 | 0.484 | 0.073 | 0.059 | 0.054 | 0.043 | 0.152 |
| | sDTI after Gibbs ringing removal | 0.393 | 0.224 | 0.109 | 0.242 | 0.528 | 0.080 | 0.049 | 0.054 | 0.035 | 0.156 |
| | BSD after Gibbs ringing removal | 0.375 | 0.226 | 0.125 | 0.242 | 0.480 | 0.071 | 0.047 | 0.049 | 0.035 | 0.141 |
| | sDTI denoised | 0.315 | 0.226 | 0.165 | 0.236 | 0.317 | 0.029 | 0.036 | 0.038 | 0.026 | 0.112 |
| | BSD denoised | 0.304 | 0.230 | 0.174 | 0.236 | 0.278 | 0.026 | 0.037 | 0.037 | 0.027 | 0.104 |
| | sDTI denoised, after Gibbs ringing removal | 0.320 | 0.225 | 0.162 | 0.236 | 0.331 | 0.048 | 0.034 | 0.042 | 0.030 | 0.123 |
| | BSD denoised, after Gibbs ringing removal | 0.310 | 0.228 | 0.171 | 0.236 | 0.295 | 0.045 | 0.034 | 0.041 | 0.031 | 0.112 |
| cc | sDTI | 0.416 | 0.232 | 0.105 | 0.251 | 0.555 | 0.092 | 0.068 | 0.061 | 0.047 | 0.173 |
| | BSD | 0.396 | 0.237 | 0.124 | 0.252 | 0.501 | 0.082 | 0.064 | 0.058 | 0.046 | 0.162 |
| | sDTI after Gibbs ringing removal | 0.418 | 0.252 | 0.121 | 0.264 | 0.512 | 0.097 | 0.064 | 0.061 | 0.049 | 0.156 |
| | BSD after Gibbs ringing removal | 0.400 | 0.255 | 0.139 | 0.265 | 0.460 | 0.089 | 0.058 | 0.057 | 0.048 | 0.148 |
| | sDTI denoised | 0.348 | 0.246 | 0.146 | 0.247 | 0.401 | 0.059 | 0.052 | 0.065 | 0.043 | 0.158 |
| | BSD denoised | 0.334 | 0.246 | 0.164 | 0.248 | 0.345 | 0.057 | 0.051 | 0.063 | 0.043 | 0.151 |

| | | | | | | | | | | | |
|---|---|---|---|---|---|---|---|---|---|---|---|
| | sDTI denoised, after Gibbs ringing removal | 0.368 | 0.254 | 0.156 | 0.259 | 0.399 | 0.068 | 0.059 | 0.074 | 0.043 | 0.191 |
| | BSD denoised, after Gibbs ringing removal | 0.353 | 0.256 | 0.174 | 0.261 | 0.345 | 0.062 | 0.057 | 0.067 | 0.044 | 0.176 |
| ec | sDTI | 0.405 | 0.227 | 0.105 | 0.246 | 0.561 | 0.089 | 0.081 | 0.073 | 0.063 | 0.171 |
| | BSD | 0.396 | 0.228 | 0.116 | 0.247 | 0.530 | 0.087 | 0.080 | 0.072 | 0.063 | 0.171 |
| | sDTI after Gibbs ringing removal | 0.392 | 0.240 | 0.117 | 0.249 | 0.509 | 0.087 | 0.074 | 0.068 | 0.056 | 0.168 |
| | BSD after Gibbs ringing removal | 0.383 | 0.241 | 0.128 | 0.251 | 0.479 | 0.082 | 0.073 | 0.066 | 0.057 | 0.162 |
| | sDTI denoised | 0.334 | 0.222 | 0.155 | 0.237 | 0.380 | 0.061 | 0.060 | 0.073 | 0.052 | 0.190 |
| | BSD denoised | 0.332 | 0.225 | 0.159 | 0.239 | 0.364 | 0.061 | 0.061 | 0.073 | 0.053 | 0.189 |
| | sDTI denoised, after Gibbs ringing removal | 0.331 | 0.231 | 0.158 | 0.240 | 0.361 | 0.065 | 0.060 | 0.065 | 0.051 | 0.165 |
| | BSD denoised, after Gibbs ringing removal | 0.328 | 0.233 | 0.164 | 0.242 | 0.342 | 0.063 | 0.060 | 0.065 | 0.051 | 0.163 |
